# DeepSeek-assisted LI-RADS classification: AI-driven precision in hepatocellular carcinoma diagnosis

**DOI:** 10.1097/JS9.0000000000002763

**Published:** 2025-06-20

**Authors:** Jun Zhang, Jinpeng Liu, Mingyang Guo, Xin Zhang, Wenbo Xiao, Feng Chen

**Affiliations:** a Department of Radiology, The First Affiliated Hospital, Zhejiang University School of Medicine, Hangzhou, Zhejiang Province, P.R. China; b Department of Radiology, Affiliated Xiaoshan Hospital, Hangzhou Normal University, Hangzhou, Zhejiang Province, P.R. China; c GE Healthcare, Shanghai, P.R. China

**Keywords:** artificial intelligence, diagnostic imaging, hepatocellular carcinoma, liver imaging reporting and data system

## Abstract

**Background:**

The clinical utility of the DeepSeek-V3 (DSV3) model in enhancing the accuracy of Liver Imaging Reporting and Data System (LI-RADS, LR) classification remains underexplored. This study aimed to evaluate the diagnostic performance of DSV3 in LR classifications compared to radiologists with varying levels of experience and to assess its potential as a decision-support tool in clinical practice.

**Materials and methods:**

A dual-phase retrospective-prospective study analyzed 426 liver lesions (300 retrospective, 126 prospective) in high-risk hepatocellular carcinoma (HCC) patients who underwent magnetic resonance imaging or computed tomography. Three radiologists (one junior, two seniors) independently classified lesions using LR v2018 criteria, while DSV3 analyzed unstructured radiology reports to generate corresponding classifications. In the prospective cohort, DSV3 processed inputs in both Chinese and English to evaluate language impact. Performance was compared using chi-square test or Fisher’s exact test, with pathology as the gold standard.

**Results:**

In the retrospective cohort, DSV3 significantly outperformed junior radiologists in diagnostically challenging categories: LR-3 (17.8% vs. 39.7%, *P* < 0.05), LR-4 (80.4% vs. 46.2%, *P* < 0.05), and LR-5 (86.2% vs. 66.7%, *P* < 0.05), while showing comparable accuracy in LR-1 (90.8% vs. 88.7%), LR-2 (11.9% vs. 25.6%), and LR-M (79.5% vs. 62.1%) classifications (all *P* > 0.05). Prospective validation confirmed these findings, with DSV3 demonstrating superior performance for LR-3 (13.3% vs. 60.0%), LR-4 (93.3% vs. 66.7%), and LR-5 (93.5% vs. 67.7%) compared to junior radiologists (all *P* < 0.05). Notably, DSV3 achieved diagnostic parity with senior radiologists across all categories (*P* > 0.05) and maintained consistent performance between Chinese and English inputs.

**Conclusion:**

The DSV3 model effectively improves diagnostic accuracy of LR-3 to LR-5 classifications among junior radiologists. Its language-independent performance and ability to match senior-level expertise suggest strong potential for clinical implementation to standardize HCC diagnosis and optimize treatment decisions.

## Introduction

The Liver Imaging Reporting and Data System (LI-RADS, LR), developed by the American College of Radiology, provides a standardized approach for hepatocellular carcinoma (HCC) imaging across screening, diagnosis, and treatment monitoring^[1]^. This system employs a comprehensive framework that classifies liver lesions based on primary and secondary imaging features, facilitates malignancy probability assessment, and guides subsequent imaging decisions^[2]^. While LR aims to standardize radiological reporting and improve data integration^[3]^, the persistent use of free-text reporting formats poses challenges for automated data extraction and contributes to interpretation variability among healthcare providers^[4]^.

Recent advancements in medical informatics have highlighted the potential of artificial intelligence to transform unstructured medical data into structured formats, particularly large language models (LLMs)^[5]^. The emergence of LLMs has significantly advanced natural language processing capabilities, attracting substantial interest across medical specialties^[6]^. In radiology, applications of GPT-4 have demonstrated promising results in various clinical tasks, including diagnostic support and report verification^[7-11]^.

Despite these developments, limited research has been conducted on LLM applications for LR classification through free-text report analysis, with existing studies showing inconsistent outcomes. Matute-González *et al*^[12]^ reported a moderate agreement between the LiverAI model and reference standards for LR classification. While Gu *et al*^[13]^ observed 85% accuracy with GPT-4 for LR assessment, Fervers *et al*^[14]^ found the suboptimal performance of ChatGPT in German radiology reports. Notably, previous investigations have primarily concentrated on assessing LLM-radiologist concordance rates while neglecting to evaluate the potential enhancement of radiologists’ diagnostic performance through LLM-assisted approaches. Moreover, the scope of existing research has been predominantly confined to ChatGPT and LiverAI models, thereby overlooking the exploration of more advanced state-of-the-art developments. In particular, the clinical interpretation of LR-2 to LR-4 categorized lesions, which represent a diagnostic grey zone encompassing both HCC and non-HCC entities. This diagnostic uncertainty significantly complicates the surgical decision-making processes, potentially resulting in treatment delays that could adversely affect patient outcomes.

Remarkably, the DeepSeek-V3 (DSV3) model, an LLM launched by DeepSeek AI in December 2024, is distinguished by its efficient training process, high performance, and cost-effectiveness. This innovation demonstrates improved training efficiency, enhanced performance metrics, and cost-effective implementation. Nevertheless, its efficacy in augmenting radiologist accuracy in LR classification requires further validation.

Therefore, our study pioneered the application of the DSV3 model in analyzing radiology reports across practitioners with diverse levels of experience to establish clinically relevant LI-RADS classifications. By systematically correlating these imaging-based categorizations with histopathological findings, we aimed to identify specific radiologist subgroups that derive maximal benefits from DSV3-assisted interpretation and optimize the integration of this technology into clinical workflows to enable stratified HCC management and precision treatment strategies. This investigation was conducted within the ethical and methodological framework established by TITAN 2025^[15]^.

**HIGHLIGHTS**

- The DeepSeek-V3 large language model demonstrated diagnostic performance comparable to that of senior radiologists (*P* > 0.05) and significantly outperformed junior radiologists (*P* < 0.05) in the classification of LI-RADS categories 3–5 lesions.
- The model significantly reduced the proportion of HCC cases classified as LI-RADS 3 (*P* < 0.05) while concurrently increasing the proportion of HCC cases classified as LI-RADS 4 (*P* < 0.05), thereby enhancing the precision of HCC risk stratification and optimizing subsequent clinical management decisions.

## Materials and methods

### Ethical approval and data privacy

The study protocol was approved by the Clinical Research Ethics Committee. Informed consent was waived. Data privacy was rigorously maintained in full compliance with the Health Insurance Portability and Accountability Act (HIPAA) guidelines and regulations^[16]^. This study was reported in accordance with the STARD (Standards for the Reporting of Diagnostic) criteria.

### Study design

This study was conducted in three phases. Firstly, liver lesions were independently assessed by radiologists with varying levels of expertise, including one junior radiologist and two senior radiologists, following LR v2018 guidelines. Secondly, the chief physician extracted relevant information from radiological text reports, which were then input into the DSV3 model for analysis. Finally, perform prospective preoperative assessment and AI-validated evaluation of selected liver lesions.

LR-1, LR-5, and LR-M were used to definitively diagnose benign lesions and HCC and non-HCC malignancies, respectively. This study evaluated the diagnostic performance of radiologists across different expertise levels and compared their results with those generated by the DSV3 model, using pathological findings as the gold standard. The primary objective was to determine whether the DSV3 model could enhance the accuracy of radiologists in classifying liver lesions.

For LR-2, LR-3, and LR-4, which indicate low, moderate, and high probabilities of HCC, respectively, this study aimed to assess whether the DSV3 model could reduce the HCC proportions in LR-2 and LR-3 while increasing it in LR-4. In clinical practice, LR-2 and LR-3 lesions are typically monitored, whereas LR-4 lesions are often subjected to biopsy or surgery to avoid treatment delays.

### Patients’ enrollment

Patients with liver lesions were identified from the institutional database based on the following eligibility criteria:

1. Magnetic resonance imaging (MRI) or computed tomography (CT) examinations were conducted from January to March 2024 for retrospective analysis, followed by a prospective study from May 1 to 20 May 2025, in compliance with LR technical guidelines.
2. Patients classified as high-risk for HCC according to LR criteria^[1]^.
3. Availability of postoperative pathological results and no prior treatment before MRI or CT scans.
4. High-quality MRI or CT images free of artifacts.

### Assessment of radiologists’ performance

Five independent radiologists with varying levels of expertise participated in the study. These included one junior radiologist (5 years of experience), two senior radiologists (15 and 20 years of experience, respectively), and two chief physicians (30 years of experience, serving as research coordinator 1 and 2 [RC 1, RC2]). One data inputter (RC 3), who was proficient in English and LLMs, participated in the study.

RC 1 extracted all liver MRI or CT examination numbers and recorded them in an Excel spreadsheet, marking the locations of the liver lesions to ensure accurate assessment, particularly in cases with multiple lesions. The largest lesion was prioritized for evaluation. Three radiologists (one junior radiologist and two senior radiologists) blinded to the pathological results independently assessed the lesions using the LR v2018 criteria in the picture archiving and communication system. To evaluate the repeatability, each radiologist performed independent assessments on three separate occasions. Final evaluations were determined based on consistency, requiring at least two or three repeated measurements to confirm the outcome. RC 2 supervised the process to ensure consistency in lesion evaluation and extracted relevant information. In the prospective study, the RC1 documented all preoperative MRI and CT examination identifiers for patients scheduled for liver surgery, in strict adherence to the previously described standardized protocol. The imaging evaluation results were compared with the postoperative pathological findings.

### Assessment of DSV3 model’s performance

The DSV3 model was accessed at https://chat.deepseek.com. RC 2 compiled the extracted information, excluding diagnostic opinions and personal details, and forwarded it to RC 3, which input the data into the DSV3 model. To minimize bias due to the model’s context sensitivity, the chat sessions were restarted, and a response was received after each report was entered. Repeatability was assessed by conducting these procedures three times for each case, with diagnoses generated two or three times, defined as the DSV3 model’s final diagnoses. In the prospective study, the model’ results were compared with the postoperative pathological findings.

### Prompts

To simulate real-world clinical applications, a zero-shot prompt was used to introduce the LR classification. For example, the prompt stated, “Your task is to apply the LR v2018 guidelines to classify the radiological findings provided. The findings are as follows:” (The content input into the DSV3 model was in Chinese and translated into English for clarity). In the prospective cohort, the DSV3 model was deployed in dual-language versions (Chinese and English) to ensure cross-regional applicability.

## Statistical analysis

Continuous variables are presented as mean ± standard deviation for normally distributed data or median (interquartile range [IQR]) for non-parametric distributions, following appropriate normality testing. Diagnostic performance comparisons between radiologists and the DSV3 model were evaluated across LR classifications (LR-1, LR-5, and LR-M) using the chi-square test or Fisher’s exact test. The diagnostic yield of HCC identification within indeterminate classifications (LR-2, LR-3, and LR-4) was systematically compared between human readers and the DSV3 model using a similar statistical methodology. Prospective validation confirmed consistent applicability of the established statistical approach. All statistical computations were performed using R statistical software (version 4.4.2; R Foundation for Statistical Computing, Vienna, Austria), with a two-tailed *P*-value threshold of <0.05 defining statistical significance.

## Results

### Study population and lesion characteristics

The study cohort comprised 300 histopathologically confirmed liver lesions, including HCC (n = 115), Intrahepatic cholangiocarcinoma (n = 45), Hepatocellular nodular hyperplasia (n = 38), Hepatocellular adenoma (n = 20), Focal nodular hyperplasia (n = 2), Hepatic hemangiomas (n = 28), and Hepatic cysts (n = 52). The imaging modalities included 178 MRI examinations and 122 CT examinations. The study population consisted of 182 male and 118 female patients, with a mean age of 54.6 years ± 12.2. The mean lesion diameter was 43.4 mm ± 16.1 (range: 12–85 mm).

**Figure 1.**
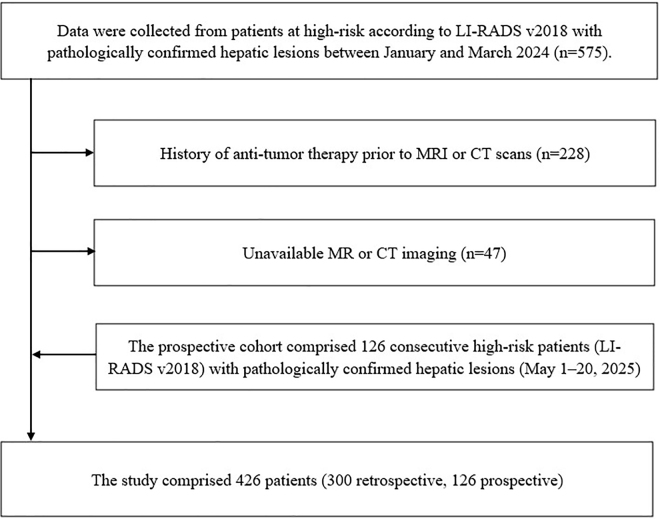
Flowchart of patient enrollment.

**Figure 2.**
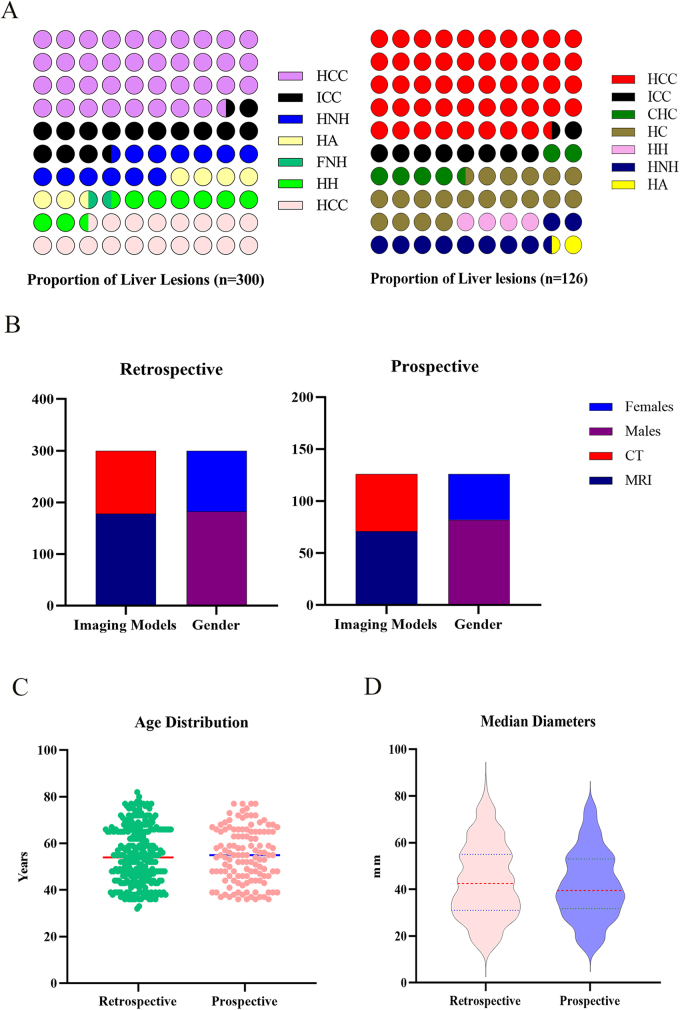
Study population and lesion characteristics. (A) Proportional distribution of liver lesions in the retrospective cohort (n = 300) and prospective validation cohort (n = 126). (B) Age distribution of patients. (C) Imaging modalities (MRI/CT) and gender distribution. (D) Mean diameters of lesions. HCC, hepatocellular carcinoma; ICC, intrahepatic cholangiocarcinoma; CHC, combined hepatocellular-cholangiocarcinoma; HA, hepatocellular adenoma; FNH, focal nodular hyperplasia; HH, hepatic hemangioma; HC, hepatic cyst; HNH, hepatocellular nodular hyperplasia; MRI, magnetic resonance imaging; CT, computed tomography.

The prospective validation cohort comprised 126 consecutively enrolled patients with histologically confirmed hepatic lesions distributed as follows: HCC (n = 61), Intrahepatic cholangiocarcinoma (n = 12), Combined hepatocellular-cholangiocarcinoma (n = 8), Hepatic cysts (n = 25), Hepatic hemangiomas (n = 5), Hepatocellular nodular hyperplasia (n = 13), and Hepatocellular adenoma (n = 2). Cross-sectional imaging modalities included contrast-enhanced MRI (71/126, 56.3%) and multiphase CT (55/126, 43.7%). Demographic analysis revealed a cohort with male predominance (82 males, 44 females; ratio 1.86:1) and a mean age of 54.7 ± 11.6 years (range: 28–82 years). Lesion size quantification showed a mean maximal diameter of 41.5 ± 14.7 mm (range: 15–76 mm). The patient enrollment flowchart and details are shown in Figs. 1 and 2, respectively.

### Performance comparison in LR-1 classification

For LR-1 classification, the diagnostic performance demonstrated no statistically significant differences between radiologists and the DSV3 model (all *P* > 0.05). The classification accuracy rates were as follows: junior radiologist (88.7% [55/62] vs. DSV3 model [90.8%, 59/65]), senior radiologist 1 (95.2% [60/63] vs. DSV3 model [96.8%, 60/62]), and senior radiologist 2 (96.8% [60/62] vs. DSV3 model [95.2%, 60/63]).

### Performance comparison in LR-2 classification

In the LR-2 classification, no statistically significant differences were observed between the radiologists and the DSV3 model (all *P* > 0.05). The classification rates were as follows: junior radiologists had a rate of 25.6% (10/39), compared to 11.9% (5/42) for the DSV3 model. The diagnostic performance of the senior radiologists was comparable, with rates of 8.1% (3/37) and 5.7% (2/35) for senior radiologists 1 and 2, respectively, versus 9.1% (3/33) and 5.4% (2/37) for the DSV3 model.

### Performance comparison in LR-3 and LR-4 classifications

Significant disparities in classification were observed between the junior radiologist and DSV3 model for both the LR-3 and LR-4 categories (*P* < 0.05). The junior radiologist demonstrated classification rates of 39.7% (23/58) for LR-3 and 46.2% (24/52) for LR-4, in contrast with the DSV3 model’s 17.8% (8/45) and 80.4% (37/46), respectively. Conversely, both senior radiologists showed no statistically significant differences compared with the DSV3 model (*P* > 0.05). For LR-3 classifications, senior radiologist 1 achieved 27.9% (12/43), while senior radiologist 2 achieved 16.7% (8/48), compared to the DSV3 model’s 23.4% (11/47) and 16.0% (8/50), respectively. Similarly, for the LR-4 classification, the diagnostic performance was comparable: senior radiologist 1 demonstrated 71.9% (41/57) versus the DSV3 model’s 70.7% (41/58), and senior radiologist 2 achieved 76.9% (40/52) compared with the DSV3 model’s 76.4% (42/55).

### Performance comparison in LR-5 classification

For the LR-5 classification, a statistically significant disparity in diagnostic accuracy was observed between the junior radiologist and DSV3 model (66.7% [40/60] vs. 86.2% [50/58], *P* < 0.05). In contrast, both senior radiologists demonstrated performance comparable to that of the DSV3 model, with no statistically significant differences (senior radiologist 1:87.9% [51/58] vs. 87.1% [54/62]; senior radiologist 2:90.9% [60/66] vs. 93.4% [57/61]; all *P* > 0.05).

### Performance comparison in LR-M classification

For the LR-M classification, no statistically significant differences in diagnostic accuracy were detected between the radiologists and the DSV3 model (all *P* > 0.05). The junior radiologist demonstrated a classification accuracy of 62.1% (18/29) compared to the DSV3 model’s 79.5% (35/44). Similarly, both senior radiologists showed comparable diagnostic performance: senior radiologist 1 achieved 88.1% accuracy (37/42) versus the DSV3 model’s 89.5% (34/38), while senior radiologist 2 demonstrated 91.9% accuracy (34/37) compared to the DSV3 model’s 91.2% (31/34). Misclassified cases are presented in Supplementary Table 1 (available at: http://links.lww.com/JS9/E418).

### Longitudinal validation

In prospective validation study, significant performance disparities were observed between junior radiologists and the DSV3 model specifically for LR-3 (60.0% [9/15] vs 13.3% [2/15], *P* < 0.05), LR-4 (66.7% [20/30] vs 93.3% [28/30], *P* < 0.05), and LR-5 (67.7% [21/31] vs 93.5% [29/31], *P* < 0.05) classifications, while no significant differences were found for LR-1 (86.7% [26/30] vs 96.7% [29/30], *P* > 0.05), LR-2 (30.0% [3/10] vs 10.0% [1/10], *P* > 0.05), or LR-M (80.0% [16/20] vs 100% [20/20], *P* > 0.05) categories. Importantly, both senior radiologists demonstrated comparable performance to DSV3 across all LR classifications (all *P* > 0.05), with these prospective findings fully consistent with our previous retrospective study conclusions (see Supplementary Table 2, available at: http://links.lww.com/JS9/E419, for complete data). Discordant cases are presented in Supplementary Table 3 (available at: http://links.lww.com/JS9/E420). The LR classification system generated identical diagnostic outputs for both Chinese and English input modalities (Supplementary Figures 1–6, available at: http://links.lww.com/JS9/E421).

**Figure 3.**
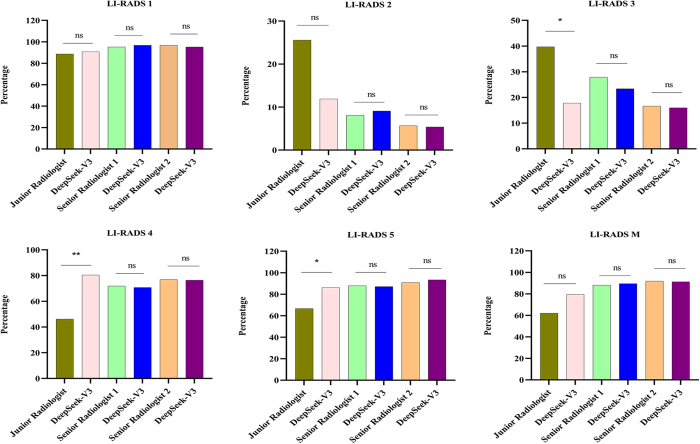
Comparison of LI-RADS classifications between radiologists with varying levels of expertise and the DeepSeek-V3 model. Significant differences are observed in LI-RADS 3–5 classifications between junior radiologists and the DeepSeek-V3 model in this retrospective analysis.

**Figure 4.**
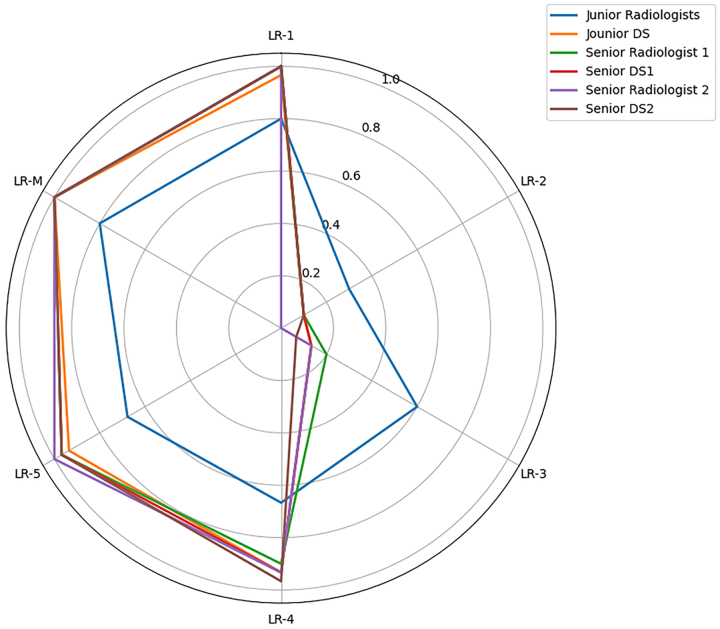
Prospective evaluation of DeepSeek-V3 versus radiologists with varying levels of expertise in LI-RADS Classification. This radar chart prospectively compares the diagnostic performance of DeepSeeK-V3 against junior and senior radiologists in classifying liver observations using LI-RADS v2018 categories. Data were obtained from a prospective cohort of 126 histologically-confirmed liver lesions, with findings concordant with previous retrospective analysis.

**Figure 5.**
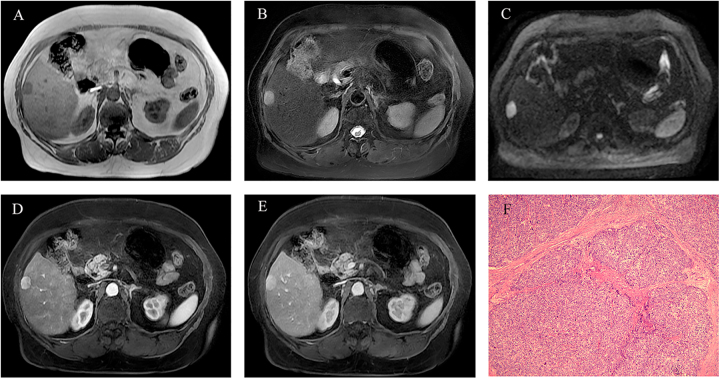
Illustrates a nodular abnormal signal in the right hepatic lobe, showing hypointensity on T1WI (A), hyperintensity on T2WI (B), and restricted diffusion on DWI (C). Contrast-enhanced imaging revealed significant arterial phase enhancement (D) with persistent enhancement in the portal venous phase (E). Histopathological examination following surgical intervention confirmed well-differentiated hepatocellular carcinoma (F).

**Figure 6.**
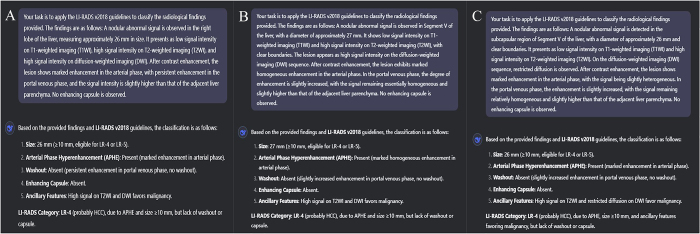
Case example demonstrating improved risk stratification by AI-assisted LI-RADS classification. (A) Initial assessment by a junior radiologist categorized the lesion as LR-3 (intermediate probability of malignancy). DeepSeek-V3 upgraded up graded the classification to LR-4 (probable hepatocellular carcinoma [HCC]) by identifying key imaging features including arterial phase hyperenhancement and a lesion size >10 mm (meeting LI-RADS v2018 major criteria). (B, C) Two senior radiologists independently categorized the lesion as LR-4, corroborating the AI’s assessment. Subsequent surgical pathology confirmed HCC. This case demonstrates the model’s capability to identify subtle imaging features indicative of early HCC, particularly valuable for assisting junior radiologists in clinical decision-making.

**Figure 7.**
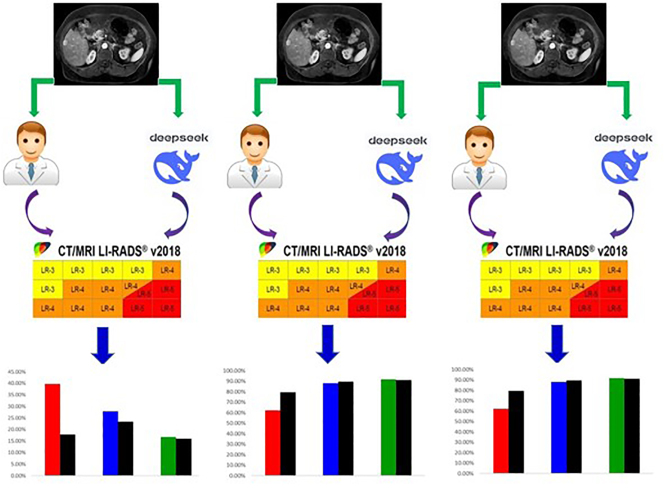
The graphical abstract presents the overall framework and workflow for comparing the diagnostic performance of the DeepSeek-V3 model and radiologists with varying expertise in LI-RADS classification.

The performance of DSV3 in LR classification was compared with that of radiologists across varying levels of expertise (Fig. 3), with further validation through prospective assessment (Fig. 4). Representative cases are shown in Figs 5 and 6. Fig. 7 presents a graphical abstract summarizing the study design and workflow of the comparison between the DSV3 model and radiologists with different levels of experience in LI-RADS classification.

## Discussion

This proof-of-concept study demonstrated the potential of the DSV3 model in generating LI-RADS classifications from free-text MRI/CT reports across radiologists with varying expertise levels. Notably, the DSV3 model significantly enhanced the classification accuracy for LR-3 to LR-5 categories among junior radiologists, while demonstrating comparable performance to senior radiologists. These findings underscore the potential of the model as a valuable decision-support tool in clinical practice, particularly for less experienced practitioners, potentially reducing diagnostic errors and improving confidence in complex cases.

The integration of LLMs into HCC imaging has undergone extensive exploration^[17,18]^. While previous investigations utilizing ChatGPT and similar LLMs for LI-RADS classification have yielded inconsistent results^[12-14]^, our study revealed distinct advantages of the DSV3 architecture. The model demonstrated superior performance to junior radiologists in the LR-3 to LR-5 classifications while maintaining comparable accuracy in the LR-1, LR-2, and LR-M categories. Its performance also matched that of the senior radiologists. These findings align with the established literature, emphasizing the critical role of clinical expertise^[19]^ and input quality^[10]^ in diagnostic accuracy. The inherent inter-observer variability among radiologists in text report generation likely contributes to output discrepancies, highlighting the need for standardized reporting frameworks. Importantly, the DSV3 model significantly improved junior radiologists’ diagnostic precision in clinically critical LR-3 to LR-5 classifications, reducing HCC prevalence in LR-3 lesions (minimizing treatment delays), while enhancing detection in LR-4/5 categories (ensuring timely intervention).

Therefore, the DSV3 model can be integrated into clinical practice through two primary approaches: as a real-time decision support tool providing immediate LR classification guidance (particularly useful for junior radiologists in differentiating ambiguous cases like LR-3 vs. LR-4 lesions) or as a second-reader system performing preliminary lesion classification to enhance workflow efficiency in high-volume centers and resource-limited settings. The model demonstrates versatile clinical utility across different experience levels, reducing diagnostic variability and serving as a training reference for junior radiologists while functioning as efficiency-enhancing support and quality control for senior radiologists, with particular value in resource-constrained environments where its pre-screening capability can help prioritize cases and compensate for limited specialist availability, allowing institutions to tailor implementation based on their specific clinical needs and available expertise.

The current literature presents conflicting evidence regarding the performance of LLM in radiological applications. While Horiuchi *et al*^[20]^ reported ChatGPT’s underperformance in complex neuroradiology cases, Suh *et al*^[10]^ observed the superior performance of junior faculty over LLMs. Conversely, Gertz *et al*^[8]^ demonstrated GPT-4’s comparable error detection capabilities to radiologists. We postulate that these discrepancies may reflect fundamental architectural differences between models. Unlike previous studies utilizing transformer-based architectures, our investigation employed the DSV3 model’s novel mixture-of-experts framework, potentially explaining its enhanced clinical performance.

Recent studies have highlighted the impact of lesion characteristics on classification accuracy. Gu *et al*^[13]^ achieved 85% accuracy using GPT-4 for lesions >21 mm, whereas Matute-Gonzalez *et al*^[12]^ reported moderate agreement for smaller lesions (<20 mm). Our findings align with these observations, with senior radiologists achieving 87-91% accuracy for LR-5 and LR-M classifications in our cohort (mean lesion diameter: 43.4 mm). This likely reflects real-world diagnostic patterns, as symptomatic presentation typically occurs in larger lesions. The diagnostic challenges associated with smaller lesions may have contributed to inconsistent findings across studies, warranting further investigation.

The effect of linguistic factors on LLM performance remains a critical consideration. Although Fervers *et al*^[14]^ reported limited ChatGPT accuracy with German reports, Gu *et al*^[13]^ achieved 85% accuracy using Korean and English inputs. Our study extends these observations to Chinese-language applications, demonstrating significant improvements in junior radiologists’ classification accuracy. Additionally, in prospective validation cohort, we addressed this fundamental concern by systematically testing each case with both Chinese and English text inputs in our model. The LR classification results demonstrated concordance between both language versions, suggesting robust performance across these linguistic contexts. However, the “black-box” nature of LLMs complicates the assessment of linguistic influences on model performance and this represents an important area for future research.

This study has several limitations that warrant consideration. First, the transmission of health information to external servers raises legitimate privacy concerns^[21-23]^. Notably, DeepSeek supports offline deployment, mitigating data privacy concerns. Second, our zero-shot approach, which is clinically pragmatic, may not represent optimal LLM utilization, given the established impact of prompt engineering on performance^[24]^. However, the DSV3 mobile interface aligns with real-world clinical workflows. Third, although the current prospective cohort study demonstrated consistent LR classification results between Chinese and English versions, further validation across additional languages remains warranted in future research. Fourth, this single-center study with a modest sample size may have inherent biases and overfitting, but our conclusions align with two recent reports publish in Nature Medicine that also employed limited single-center datasets^[25,26]^. Multicenter validation will be essential for future research. Finally, while our study benefitted from China’s standardized radiology training framework, the generalizability of the findings to other healthcare systems requires validation.

In conclusion, the DSV3 model represents a transformative advancement in radiological decision support, particularly in enhancing the diagnostic capabilities of junior radiologists for critical LR-3 to LR-5 classifications. Its ability to optimize HCC probability stratification significantly improves diagnostic precision and clinical decision-making, facilitating reliable treatment stratification and advancing precision medicine. Future multicenter studies should validate these findings and explore optimal integration strategies to maximize the clinical impact and patient outcomes.

## Data Availability

Data generated or analyzed during the study are available from the corresponding author upon request.
